# Knowledge, Attitudes, and Practices Among Parents Regarding Childhood Obesity in the United Arab Emirates: A Cross-Sectional Study

**DOI:** 10.3390/ijerph22020309

**Published:** 2025-02-18

**Authors:** Sojoud Alsheraifi, Fatima Almeleh, Hiba Rabie, Amnah Alkaabi, Sumayya AlHamrooni, Aisha Abdullah

**Affiliations:** 1 Emirates Health Services UAE, Dubai P.O. Box 2299, United Arab Emirates; fatima.almeleh@ehs.gov.ae (F.A.); hiba.mohammed@ehs.gov.ae (H.R.); amnah.binghanim@ehs.gov.ae (A.A.); sumayya.alhamrooni@ehs.gov.ae (S.A.); aisha.amohammed@ehs.gov.ae (A.A.); 2 Family Medicine Emirati Board Examination Subcommittee, Al Ain P.O. Box 15551, United Arab Emirates; 3 Family Medicine Department, University of Sharjah, Sharjah P.O. Box 27272, United Arab Emirates

**Keywords:** knowledge, attitude, practice, parents, childhood obesity

## Abstract

Obesity is a global issue whose prevalence continues to rise at a concerning rate. Over the past 30 years, many countries have witnessed the doubling or tripling of obesity rates. The growing prevalence of obesity in children is particularly worrying given that it indicates a future burden on healthcare systems. Herein, we aim to evaluate the knowledge, attitudes, and practices related to childhood obesity among parents of school children in Ras Al-Khaimah (RAK) and Fujairah, United Arab Emirates (UAE). This cross-sectional study was conducted between January and May 2024 and involved parents/guardians of children from grades 1 to 12 in governmental schools in RAK and Fujairah. A standardized questionnaire adapted from the “We Can Program” was used after modification to make it suitable for the study participants. The appropriate sample size was calculated and found to be 471. A total of 510 questionnaires were included in the analysis. The study participants comprised 89.6% females, 77.6% middle-aged individuals, and 61.8% RAK residents. The results show that 96.3% of parents have good knowledge; 88.4% have a positive attitude; and 97.8% have good practices. Younger and less educated parents have a less positive attitude {odds ratio (OR) = 0.052 (0.28–0.98), *p* = 0.03 and OR = 0.057 (0.33–1.009), *p* = 0.03, respectively}. In contrast, having good knowledge increases the chances of having a positive attitude {OR = 3.81 (1.39–10.45), *p* = 0.015}. Males were found to have a lower probability of having good practices {OR = 0.29 (0.07–0.15), *p* = 0.09}. However, having good knowledge and a positive attitude increases the chances of having good practices {OR = 6.30 (1.26–31.41), *p* = 0.05 and OR = 23.42 (6.02–91.09, *p* = 0.00), respectively}. In conclusion, overall, parents/guardians from RAK and Fujairah have good knowledge, positive attitudes, and good practices with regard to childhood obesity. Young parents with lower educational levels tend to have a negative attitude. In general, living in RAK or Fujairah is not a contributing factor affecting the levels of overall knowledge, attitudes, and practices. However, parents in Fujairah have worse practices according to the majority of the individual practice questions related to physical activity and healthy food compared to parents in RAK. Particular emphasis should be placed on walking, biking, and using the stairs, when possible, among parents in Fujairah. National programs should be developed, targeting young parents with lower educational levels to improve their attitudes and hence their practices.

## 1. Introduction

Obesity is a global public health issue with alarmingly increasing prevalence. The condition is labeled as a national epidemic and affects one in three adults and one in six children in the United States of America [1]. From 1975 to 2016, the prevalence of being overweight and obese among children and adolescents aged 5–19 years increased by more than four-fold from 4% to 18% worldwide, with most of the affected individuals living in developing countries [2]. The World Health Organization (WHO) defines obesity and overweight as abnormal or excessive fat accumulation risking individual health. The state of being overweight in individuals aged 5–19 years is defined as a body mass index (BMI) ≥ 1 standard deviation (SD), and obesity is defined as a BMI ≥ 2 SD of the median for age and sex [3]. In contrast, for those aged 2–20 years, the Centers for Disease Control and Prevention (CDC) defines overweight in children as a BMI greater than the 85th percentile to less than the 95th percentile and defines childhood obesity as a body mass index greater than or equal to the 95th percentile based on CDC growth curve charts [4].

Rubino et al. 2025 mentioned that the Lancet Diabetes & Endocrinology Commission highlights the inadequacy of BMI as a sole measure of obesity as it can both overestimate and underestimate health risks. The commission proposed defining clinical obesity as a chronic disease caused by excess adiposity affecting organ and tissue function, leading to serious health complications such as heart disease, stroke, and renal failure. They distinguish preclinical obesity as excess adiposity without immediate functional impairment but with an increased risk of disease development. A diagnosis of clinical obesity should be based on either a direct measurement of body fat or additional anthropometric criteria beyond BMI [5].

The results of a study published in 2014 demonstrate that the prevalence of obesity among children and adolescents ranges from 5% to 14% in males and from 3% to 18% in females in Gulf countries. A trend of increased obesity prevalence among children and adolescents has also been noticed in studies conducted in Saudi Arabia, Qatar, Kuwait, Bahrain, and the United Arab Emirates (UAE) [6]. The latest update in 2023 from the Global Observatory of the World Obesity Federation showed that the UAE has a childhood obesity prevalence rate of 15% among girls (ranked 24th worldwide) and 18.5% among boys (ranked 21st worldwide) [7].

A population-based study from the UAE conducted between 2013 and 2015 involving 44,942 students attending governmental schools in Ras Al-Khaimah (RAK) showed a rise in child obesity rates from 3 to 12 years. One-fourth of students aged 11–14 were either obese or morbidly obese, and 10% of boys and 3% of girls aged 15–18 were morbidly obese [8]. In 2019, the prevalence rates of being overweight and obese among adolescents in schools in Dubai were 25.3% and 15%, respectively. While the prevalence of being overweight was similar between male and female students, obesity was more common among males [9].

Childhood obesity increases the risk of hypertension, insulin resistance, sleep apnea, and asthma and is related to the incidence of fatty liver and gastroesophageal reflux. Obese children struggle with social stigma and bullying and are at a higher risk of anxiety and depression [10].

Childhood and adolescent obesity is related to biological predisposition and socioeconomic and environmental factors that, together, stimulate the deposition and proliferation of adipose tissue and resistance to obesity management efforts. Families influence the creation of an obesogenic environment by modeling physical activities, food habits, sleep patterns, and screen use. Obesity is also related to early life events, including maternal factors, infant feeding practices, and social factors [11].

Research has shown that parents’ attitudes and behaviors play an important role in impacting their child’s healthy eating behaviors, physical activity levels, and sleeping patterns, with these factors possibly preventing children from becoming obese or overweight [12]. Evidence from research suggests that parents’ positive practices can lower the risk of obesity in children [13].

In their cross-sectional study, Zoghby et al. (2022) examined Lebanese parents’ knowledge, attitudes, and practices (KAP) regarding childhood obesity, highlighting the importance of parent–physician communication. Their results reveal knowledge gaps, attitudinal barriers, and inconsistent health practices, with some parents underestimating their child’s weight and failing to implement healthy habits. The authors concluded that good parental knowledge is associated with better parental attitudes and practices with regard to childhood obesity [14].

Furthermore, the results of a survey involving 1440 school children and their parents from the Abu Dhabi Childhood Obesity Study highlight the critical role of parental weight perception in the prevention and management of childhood obesity in the UAE. The authors concluded that parents’ underestimation of their children’s weight statuses results in them overlooking health advice aimed at managing obesity [15].

Childhood obesity is a growing concern in the United Arab Emirates caused by sedentary lifestyles, fast food consumption, and cultural perceptions. Climatic conditions also limit outdoor activity. Many parents underestimate obesity risks, which may aggravate the problem. Investigating parental knowledge, attitudes, and practices (KAP) is important to develop targeted interventions that address these region-specific challenges, promote healthy lifestyle choices, and reduce obesity-related health risks in children.

To our knowledge, no previous study conducted in the Northern Emirates in the UAE has assessed parents’ knowledge of, attitudes toward, and practices regarding childhood obesity. In light of the lack of research in this regard, herein, we aim to evaluate the levels of knowledge, attitudes, and practices related to childhood obesity among parents of school children in Ras Al-Khaimah (RAK) and Fujairah, UAE, and to assess some of the factors that contribute to KAP levels.

Our study’s hypothesis is that parents in RAK and Fujairah have good knowledge of, positive attitudes toward, and good practices regarding childhood obesity. We also hypothesize that parents with greater knowledge about childhood obesity exhibit more positive attitudes and good practices related to their children’s levels of nutrition and physical activity.

## 2. Materials and Methods

### 2.1. Study Design and Participants

This quantitative, descriptive, cross-sectional study was conducted between January and May 2024 to assess the knowledge, attitudes, and practices of parents/guardians of school children with regard to childhood obesity. All parents/guardians of children from grades 1 to 12 registered at all governmental schools in two cities in the UAE, namely, RAK and Fujairah, in the academic year of 2023–2024 were eligible for inclusion (including 55 schools in RAK and 35 schools in Fujairah). Any parent/guardian who was on vacation, unable to speak Arabic or English, or refused to participate in the study was excluded with no further consequences. The total number of students registered in governmental schools from grades 1 to 12 for the academic year of 2023–2024 was 55,588, with 31,585 students in RAK and 24,003 students in Fujairah.

As the total population who met our inclusion and exclusion criteria was 55,588, Epi-info software was used to estimate the minimal sample size needed. The minimum sample size was calculated as 471 with a margin of error of 4.5%, 95% confidence intervals (CI95%), and a 50% (+/−5) hypothesized frequency of outcome in the population. The final sample size obtained was 510 questionnaires.

### 2.2. Questionnaire

The questionnaire used in this study was adapted from the “We Care program” [16], with the author’s permission. It was translated into Arabic and piloted on 20 parents/guardians without modifications, ensuring both cultural relevance and reliability throughout the process. While the original content was preserved, special attention was given to ensuring that the translation not only conveyed the words accurately but also aligned with the local cultural context. The translation was performed by bilingual experts fluent in both English and Arabic, followed by a thorough review by healthcare professionals familiar with the UAE’s cultural and healthcare landscapes. The pilot study was instrumental in confirming that participants found the questions clear and culturally appropriate. No major issues were identified, which further validated the suitability of the translated version. To maintain the integrity of the questionnaire, a rigorous translation process was followed, including both forward and backward translation. This ensured that the Arabic version remained faithful to the original intent. Additionally, a panel of experts in public health and genetic counseling reviewed the questionnaire to assess its clarity and relevance. To assess reliability, a subset of participants completed the questionnaire twice to evaluate the test–retest reliability, confirming that the responses remained stable over time.

The initial section of the questionnaire includes an informed consent form, which provides parents with detailed information about the research and seeks parental approval to use the provided information for research purposes. Parents/guardians had the option to either agree or decline to participate. Note that no monetary reward was given to those who chose to participate. The questionnaire consists of four main parts. The first part of the questionnaire includes parent-related demographic data, namely, age, gender, level of education, job, total family monthly income, and emirate of residency. The second part consists of eight questions related to the knowledge of parents/guardians regarding healthy food, physical activity, energy expenditure, and the screen time of their children. The knowledge questions have true or false choices as answers. The third part of the questionnaire assesses the attitudes of parents/guardians and contains 10 questions with five choices ranging from strongly disagree to strongly agree based on the Likert scale. The scores given for these questions range from 1 to 5 (1 = strongly disagree; 5 = strongly agree). The fourth part of the questionnaire assesses parents/guardians’ practices regarding childhood obesity and contains 14 questions with five choices ranging from strongly disagree to strongly agree based on the Likert scale. The scores given for the questions range from 1 to 5 (1 = strongly disagree; 5 = strongly agree). For a further assessment of the levels of knowledge, attitudes, and practices, eight questions were asked to analyze the parents’ level of knowledge, with true or false choices as answers. The correct answer was scored 1, whereas the wrong answer was scored 0, with a maximum possible score of 8. Respondents who achieved a score of 5 or higher were labeled as having good knowledge, while those who scored less than 5 points were labeled as having poor knowledge. Ten attitude-related questions were used, with five choices ranging from strongly disagree (score of 1) to strongly agree (score of 5) for a positive attitude based on the Likert scale with a maximum possible score of 50. Respondents who achieved a score of more than 35 points were labeled as having a positive attitude, whereas those who scored 35 or less were labeled as having a negative attitude. Lastly, 14 practice questions with five choices were asked, with answers ranging from strongly disagree (score 1) to strongly agree (scored 5) for good practices based on the Likert scale, with a maximum possible score of 70. Respondents who achieved a score of 42 and higher were labeled as having good practices, whereas those who scored less than 42 were labeled as having bad practices.

For more details about the questions in part 2, 3, and 4, kindly refer to the Supplementary Materials.

### 2.3. Data Collection and Ethical Approval

Before the start of the data collection phase, all governmental schools in RAK and Fujairah were contacted by the research authors by email, and agreement was secured from the heads of all government schools in Ras Al-Khaimah (RAK) and Fujairah to participate in the study, with the exception of one school (A. B. School) that refused to participate.

The registered parents’ mobile phone numbers were taken from the school parents’ registry. The questionnaire was distributed using the contact numbers of all registered parents within the school database through the Telegram messaging platform by the assigned school nurse. Telegram serves as the official communication platform between schools and parents in UAE government schools. As the response rate from parents was poor at the beginning of the study, several reminders were sent. Once the required number of participants was achieved, the questionnaire was closed so that no more questionnaires could be completed.

Participants had the choice not to complete the survey with no further consequences. Consent was obtained before the survey. The initial section of the questionnaire includes an informed consent form, which provides parents with detailed information about the research and seeks parental approval to use the provided information for research purposes. Data confidentiality was ensured by using an anonymous questionnaire. Moreover, only the study authors had access to the data. The study proposal was approved by the Ministry of Health and Prevention Research Ethical Committee under approval number MOHAP/DXB-REC/J.F.M/No. 24/2024.

### 2.4. Statistical Analysis

Qualitative data were categorized to identify the key results and patterns. All of the quantitative data analyses were performed using the Statistical Package for the Social Sciences or SPSS software version 23. Statistical significance was determined using a *p*-value level < 0.05. To determine the effect of demographic factors, such as the parents’ age or gender, on the levels of knowledge, attitudes, and practices, Chi square and odds ratios (ORs) (95% CI) were calculated.

## 3. Results

A total of 510 questionnaires about childhood obesity were completed and submitted by the parents/guardians of school children in RAK and Fujairah.

### 3.1. Sociodemographic Characteristics

The sociodemographic characteristics of the research population are displayed in Table 1, with the results demonstrating that, of the study sample, 89.6% are female; 77.6% are aged between 31 and 50; 65.3% of families have only one working parent; and 61.8% are residents of RAK. Regarding the level of education, 46.3% of the study population have university degrees; 41.6% have secondary school degrees; 7.5% have not achieved their secondary school certificates; and only 4.7% have postgraduate certificates. Lastly, 42% of the study sample have an average of AED 15,000–25,000 as their total family monthly income, followed by 35.1% with a total family monthly income of AED > 25,000 and only 22.9% having less than AED 15,000 as their total family monthly income.

### 3.2. Knowledge of, Attitudes Toward, and Practices Regarding Childhood Obesity Among the Study Population

Table 2 summarizes the primary outcome of this study. From the results, it can be seen that 96.3% of the parents/guardians of the school children in the governmental schools in RAK and Fujairah have good knowledge; 88.4% have a positive attitude; and 97.8% have good practices regarding childhood obesity.

For more details about the individual questions used in the questionnaire, please refer to the Supplementary Materials.

### 3.3. Factors Affecting the Knowledge, Attitude, and Practice Levels Regarding Childhood Obesity Among the Study Population

The results presented in Table 3 show the different factors affecting the knowledge, attitude, and practice (KAP) levels regarding childhood obesity among parents/guardians of school children in governmental schools in RAK and Fujairah. From the results, it can be seen that younger and less educated parents/guardians have reduced chances of having a positive attitude toward childhood obesity {OR = 0.52 (0.28–0.98), *p* = 0.03 and OR = 0.57 (0.33–0.98), *p* = 0.03, respectively}. In contrast, having good knowledge increases the chances of having a positive attitude toward childhood obesity {OR = 3.81 (1.39–10.45), *p* = 0.015}.

The results displayed in Table 3 also show that having good knowledge and a positive attitude increases the chances of having good practices {OR = 6.30 (1.26–31.41), *p* = 0.049 and OR = 23.42 (6.02–91.09), *p* = 0.00, respectively}. All other factors did not show any clinically or statistically significant relationships.

### 3.4. Effect of Emirate of Residency on Positive Attitudes and Good Practices Among Parents/Guardians Regarding Childhood Obesity

The results displayed in Table 4 show that there was no difference in positive attitudes toward having “no safe or convenient place for family to be physically active” in terms of residency in either Fujairah or RAK. In contrast, parents/guardians from Fujairah had a less positive attitude toward time available for physical activity (“There is no enough time in the day to do physical activity”) compared with parents/guardians from RAK {OR = 0.58 (0.34–0.99), *p* = 0.03}.

Regarding practices, parents/guardians from Fujairah implement fewer good practices toward the following: “If I am physically active, there is a good chance my family will follow my example”, “Whenever I can, I walk or bike places instead of driving”, “I use the stairs instead of the elevator when I can”, “When eating foods that are high in fat, I try to keep the portions small”, and “I often monitor the portion size of food served to my family” {OR = 0.14 (0.03–0.50), *p* = 0.001; 0.51 (0.32–0.81), *p* = 0.004; 0.42 (0.22–0.83), *p* = 0.009; 0.377 (0.20–0.70), *p* = 0.003; and 0.58 (0.36–0.93), *p* = 0.018, respectively}.

## 4. Discussion

Several recommendations, such as improving the environment where the children live, implementing policies for developing a healthy food environment, ensuring an accessible, safe environment for promoting physical activity in children, and easy access to health facilities for obesity prevention and treatment services, have been proposed by the WHO Commission on Ending Childhood Obesity (2016) to halt this rapidly growing epidemic worldwide [17]. In the present study, we attempted to assess the levels of knowledge, attitudes, and practices of parents/guardians regarding childhood obesity and analyze different factors affecting parents/guardians’ KAP levels to be able to apply a national strategy to combat this important and growing public health issue. As hypothesized, we found that the percentage of parents/guardians with good knowledge, positive attitudes, and good practices regarding childhood obesity is high. This finding could indicate many positive signs, including good community awareness, good media coverage, good medical services, and the availability of national obesity-fighting programs. The UAE government has begun to address this issue, which clearly demonstrates that, according to their perspective, it is necessary to combat the problem.

When examining the factors affecting knowledge levels, in a study on mothers’ KAP levels regarding childhood obesity conducted in Malaysia by Hatta et al. in 2017 [18], it was found that a higher educational level, an older age, and higher monthly income were associated with a higher level of knowledge. However, in our study, age, level of education, and total monthly income had no effect on the level of knowledge. This contrast may be linked to the availability of information regarding childhood weight using different media/information outlets (TV, radio, social media, healthcare personnel, and many others) that suit different ages, educational levels, and economic statuses in the UAE.

Regarding attitudes, representing another important dimension investigated in this work, we conclude that a younger age and a lower educational level are associated with less positive attitudes. In contrast, Hatta et al. [18] showed in their study that those who achieved a higher educational level have better attitudes. These similar results can be explained by the fact that gaining more knowledge increases the likelihood of having a positive attitude in many aspects of life, including lifestyle and maintaining a normal weight. Younger and less educated parents might have less experience, lower knowledge acquisition, and limited access to information, and they may be more influenced by family and cultural traditions, which might lead to a variety of negative implications in the community, including underestimating and misunderstanding the seriousness of childhood obesity and being less informed about the resources and preventive strategies available, factors that may play a major role in increasing the prevalence of childhood obesity in the community.

Regarding practices, Hatta et al. [18] concluded that age, gender, level of education, and monthly income have no effect on practices. We also found that having good knowledge reflects positively on the parents’ attitudes and practices and having a positive attitude reflects positively on parents’ practices. The same finding was obtained by Garcia-Conde, who conducted a study involving 21 schools in the main cities of the Region of Murcia, Spain. They concluded that parental behaviors consistent with their attitudes show a significant influence on children’s healthy behaviors [12]. These findings help to improve parents’ knowledge about the causes of obesity, risks, and preventive measures necessary to help parents make better decisions regarding their children’s health and increase their motivation to ask for help from healthcare providers to prevent or manage childhood obesity.

Our study focused on two emirates in the UAE (RAK and Fujairah) and aimed to determine the levels of KAP in addition to the contributing factors that can reduce the prevalence of childhood obesity. We found that, in general, living in a particular emirate is not a contributing factor affecting the overall levels of KAP. However, while assessing the questions individually, we found that parents from Fujairah demonstrated less beneficial practices in the majority of the questions than parents from RAK, including being physically active and eating healthy food. Particular emphasis should be placed on walking, biking, and using the stairs, when possible, among parents from Fujairah.

Attitudes toward perceived barriers to being physically active were assessed. The lack of safe and convenient spaces for family members to undertake physical activity was not an issue in either of the emirates. This finding indicates the importance of efforts made by the UAE government to ensure the availability of such spaces that are free of charge in public parks, including walking and cycling tracks and gym equipment. However, time might be a perceived barrier to family members undertaking physical activity in Fujairah compared to RAK.

Given that this study is cross-sectional, it is not possible to establish causality or assess changes in knowledge, attitudes, and practices over time or in response to interventions or evolving societal norms. Additionally, this study did not account for cultural factors, which are important determinants of attitudes and practices regarding childhood obesity. Moreover, because the questionnaire used in this study was adopted from the “We Can Program”, it may not have been culturally validated for the UAE population, potentially affecting the reliability of the data collected. Furthermore, the reliance on self-reported data may have led parents to report more favorable attitudes and practices regarding childhood obesity prevention. Future research should incorporate longitudinal studies to monitor how parents’ knowledge, attitudes, and practices regarding childhood obesity evolve over time, particularly following public health campaigns or school-based interventions. It is also recommended that future studies examine the cultural factors that influence parents’ attitudes and practices, as well as psychological factors, such as body image perceptions, that may affect parents’ approaches to childhood obesity prevention.

## 5. Conclusions

Overall, the results of this study show that parents/guardians from RAK and Fujairah have good knowledge, positive attitudes, and good practices regarding childhood obesity. Young parents with lower educational levels tend to have a negative attitude compared to those in other age groups. As expected, parents with good knowledge have positive attitudes and good practices, and parents with a positive attitude have good practices. We can conclude that, in general, living in RAK or Fujairah was not a contributing factor affecting the overall level of KAP. However, parents in Fujairah had worse practices related to physical activity and healthy food compared to parents in RAK. We found no difference between RAK and Fujairah parents’ attitudes toward having a safe place for exercise. However, parents in Fujairah perceived time as a barrier to physical activities.

## Figures and Tables

**Table 1:** The sociodemographic characteristics of the study population (N = 510).

Character	N	(%)
Gender		
- Male	53	(10.4)
- Female	457	(89.6)
Age in years		
- <20	45	(8.8)
- 21–30	46	(9.0)
- 31–50	396	(77.6)
- >50	23	4.5
Education		
- <Secondary	38	(7.5)
- Secondary	212	(41.6)
- University	236	(46.3)
- Postgrad	24	(4.7)
Working		
- One parent	333	(65.3)
- Two parents	118	(23.1)
- None	59	(11.6)
Monthly income		
- AED < 15,000	117	(22.9)
- AED 15–25 K	214	(42.0)
- AED > 25,000	179	(35.1)
Residency		
- Fujairah	187	(36.7)
- RAK	315	(61.8)
- Other	8	(1.6)

**Table 2:** The overall results for the levels of knowledge, attitudes, and practices among the study population (N = 510).

KAP (Knowledge, Attitudes, and Practices) Levels	N	(%)
Knowledge level		
- Good	491	(96.3)
- Poor	19	(3.7)
Attitudes		
- Positive	451	(88.4)
- Negative	59	(11.6)
Practices		
- Good	499	(97.8)
- Bad	11	(2.2)

**Table 3:** Factors influencing good knowledge, positive attitudes, and good practices among the study population (N = 510).

Character	Good Knowledge		Positive Attitude		Good Practice	
	OR (CI 95%)	*p*-Value	OR (CI 95%)	*p*-Value	OR (CI 95%)	*p*-Value
Gender						
- Male	0.60 (0.17–2.14)	0.43	0.70 (0.31–1.58)	0.39	0.29 (0.07–1.15)	0.09
Age						
- < or = 30	0.58 (0.20–1.67)	0.23	0.52 (0.28–0.98)	0.03	0.36 (0.10–1.275)	0.11
Education						
- Secondary and lower	0.86 (0.34–2.16)	0.46	0.57 (0.33–0.99)	0.03	0.35 (0.09–1.35)	0.1
Monthly income:						
- AED < 25,000	0.65 (0.23–1.84)	0.29	1.03 (0.585–1.81)8	0.5	0.69 (0.18–2.642)	0.42
Residency						
- Fujairah	0.73 (0.28–1.89)	0.34	0.92 (0.52–1.61)	0.44	0.70 (0.21–2.35)	0.19
Knowledge						
- Good	-	-	3.81 (1.39–10.45)	0.015	6.30 (1.26–31.41)	0.049
Attitudes						
- Positive	-	-	-	-	23.42 (6.02–91.09)	0.00

**Table 4:** Effect of parents’ place of residency on answers given regarding positive attitudes and good practices to individual questions among study population (N = 510).

**Domain**	**Question**	**Parents’ Place of Residency**			
		**Fujairah** **(N = 187)**	**RAK** **(N = 314)**	**Fujairah vs. RAK**	
		**N (%)**	**N (%)**	**OR (CI 95%)**	***p*** **-Value**
Positive attitude answers	There is no safe or convenient place for my family to be physically active.	44/187 (23.5%)	82/314 (26.1%)	0.87 (0.57–1.32)	0.29
	There is not enough time in the day to carry out physical activity.	22/187 (11.8%)	58/314 18.5%	0.58 (0.34–0.99)	0.03
Good practice answers	If I am physically active, there is a good chance my family will follow my example.	174/187 (93.5%)	311/314 (99%)	0.14 (0.03–0.50)	0.001
	Whenever I can, I walk or bike to places instead of driving.	142/187 (75.9%)	270/314 (86%)	0.51 (0.32–0.81)	0.004
	I use the stairs instead of the elevator when I can.	165/187 (88.2%)	297/314 (94.6%)	0.42 (0.22–0.83)	0.009
	When eating foods that are high in fat, I try to keep the portions small.	161/187 (86.1%)	296/314 (94.3%)	0.377 (0.20–0.70)	0.003
	I often monitor the portion size of food served to my family.	147/187 (78.6%)	271/314 (86.3%)	0.58 (0.36–0.93)	0.018

## Data Availability

The data presented in this study are available from the corresponding author upon request. The data are not publicly available due to ethical restrictions.

## Supplementary Materials

The following supporting information can be downloaded at https://www.mdpi.com/article/10.3390/ijerph22020309/s1, Table S1: Percentages of knowledge questions correctly answered (n = 510); Table S2: Percentages of participants with positive attitudes per question (n = 510); Table S3: Percentages of participants with good practices per question (n = 510).
