# Supplementary material for: Knowledge, Attitudes, and Practices Among Parents Regarding Childhood Obesity in the United Arab Emirates: A Cross-Sectional Study
Source: Int J Environ Res Public Health. 2025 Feb 18;22(2):309. doi: 10.3390/ijerph22020309 (PMC11855547; doi:10.3390/ijerph22020309)
Supplement: Supplementary file 1 [file ijerph-22-00309-s001.zip › ijerph-3440867-supplementary.pdf]

Table S1. *Percentages of knowledge questions correctly answered (n = 510).*

Knowledge questions	N	(%)
Children should spend at least 1 h each day in moderate to vigorous intensity physical activity.	390	(76.5)
Maintaining healthy weight just by making sure that food intake equals physical activity on most days.	476	(93.3)
When a person eats larger portions, more physical activity will not prevent him/her from gaining weight.	260	(51.0)
Fried foods and baked goods should only be eaten occasionally.	469	(92.0)
Removing the skin from poultry is a good way to reduce the fat.	469	(92.0)
Children who do not have television in their bedrooms spend as much time watching television as children who do not have them in their bedrooms.	60	(11.8)
People tend to eat less when they spend a lot of time watching TV/iPad tablet.	299	(58.6)
Children in homes where rules on watching TV are enforced spend less time watching TV/iPad tablet than children in where such rules are not enforced.	299	(58.6)

Table S2. *Percentages of participants with positive attitude per question (n=510).*

Attitude questions	N	(%)
There is no safe or convenient place for my family to be physically active.	126	(24.8)
There is no enough time in the day to find time to be physically active.	81	(15.9)
I can think of several ways (other than weight control and the physical health benefits) that my family and/or I can benefit from being physically active.	429	(84.1)
I can find creative ways to be physically active.	450	(88.3)
Balancing the calories that I get from eating with moderate levels of physical activity is easy to do.	351	(68.9)
Choosing a diet without a lot of added sugar is important.	481	(94.3)
Choosing a diet low in fat is important.	431	(84.5)
Choosing a diet with plenty of fruits and vegetables.	466	(91.4)
Spending too much time watching TV, playing video games or iPad tablet could be bad for my child's health.	497	(97.4)
It is important to me to find alternatives to watching TV/ iPad tablet for my family.	493	(96.6)

Table S3. *Percentages of participants with good practice per question (n = 510).*

Practice questions	N	(%)
If I am physically active, there is a good chance my family will follow my example.	494	(96.8)
I try to be physically active most days.	498	(97.6)
Whenever I can, I walk or bike places instead of driving.	420	(82.3)
I use the stairs instead of the elevator when I can.	471	(92.3)
When eating foods that are high in fat, I try to keep the portions small.	466	(91.3)
I often monitor the portion size of food served to my family.	426	(83.5)
I play an important role in determining what my child/children eat.	451	(88.4)
I play an important role in determining how much physical activity my child/children get.	416	(81.5)
If I eat well, there is a good chance my family will follow my example.	473	(92.7)
I often make sure that healthy snacks are easily available for my family.	450	(88.2)
I often choose healthy foods for myself.	426	(83.5)
In my family, we have set some rules on foods and eating that we try to follow.	392	(76.8)
I enforce rules on screen time (watching TV, using a home computer for recreation, or playing video or electronic games) in my family's home.	408	(80)
More often than not, my family and I do not watch TV/iPad tablet during meal times.	375	(73.5)
